# Mode of Transmission Determines the Virulence of *Black Queen Cell Virus* in Adult Honey Bees, Posing a Future Threat to Bees and Apiculture

**DOI:** 10.3390/v12050535

**Published:** 2020-05-14

**Authors:** Yahya Al Naggar, Robert J. Paxton

**Affiliations:** 1 General Zoology, Institute for Biology, Martin Luther University Halle-Wittenberg, Hoher Weg 8, 06120 Halle (Saale), Germany; robert.paxton@zoologie.uni-halle.de; 2 Zoology Department, Faculty of Science, Tanta University, Tanta 31527, Egypt

**Keywords:** *Apis mellifera*, BQCV, mortality, innate immunity, RNAi, antiviral response

## Abstract

Honey bees (*Apis mellifera*) can be infected by many viruses, some of which pose a major threat to their health and well-being. A critical step in the dynamics of a viral infection is its mode of transmission. Here, we compared for the first time the effect of mode of horizontal transmission of *Black queen cell virus* (BQCV), a ubiquitous and highly prevalent virus of *A. mellifera*, on viral virulence in individual adult honey bees. Hosts were exposed to BQCV either by feeding (representing direct transmission) or by injection into hemolymph (analogous to indirect or vector-mediated transmission) through a controlled laboratory experimental design. Mortality, viral titer and expression of three key innate immune-related genes were then quantified. Injecting BQCV directly into hemolymph in the hemocoel resulted in far higher mortality as well as increased viral titer and significant change in the expression of key components of the RNAi pathway compared to feeding honey bees BQCV. Our results support the hypothesis that mode of horizontal transmission determines BQCV virulence in honey bees. BQCV is currently considered a benign viral pathogen of adult honey bees, possibly because its mode of horizontal transmission is primarily direct, per os. We anticipate adverse health effects on honey bees if BQCV transmission becomes vector-mediated.

## 1. Introduction

Honey bees (*Apis mellifera* L.) offer highly valued pollination services for a wide range of agricultural crops and rank as the world’s most important pollinator species [1,2]. They are, however, exposed to a variety of pathogens, including viruses, which pose significant threats to their health and well-being [3]. Over 24 viruses have been reported from honey bees worldwide, some of which can have dramatic effects on honey bee health [4,5,6]

Transmission, the movement of an infectious agent from one host to another, and virulence, the ability to reduce host fitness, are two fundamental biological parameters of a pathogen [7]. Intra-host replication of a pathogen links its transmission to its virulence; increasing parasite titer increases the likelihood of transmission but also increases the likelihood of host death [8], a common measure of virulence; therefore, over evolutionary time, transmission is considered to trade off against virulence [8]. Transmission processes are thought to play an important role in determining the spread and persistence of viral pathogens in honey bee populations [9].

Viruses may be transmitted vertically, horizontally, or both [7]. Horizontal transmission is usually considered to lead to increased virulence compared to vertical transmission. This is because, in vertical transmission, a pathogen’s transmission is generally linked to host longevity [8], with recent experimental support for theory seen in an isopod and its endosymbiont *Wolbachia* [10]. Within horizontal transmission, pathogens can be transmitted by one of two modes, either directly between individuals, through consumption of feces and contaminated food, or indirectly through an intermediate biological vector [11]. Over evolutionary time, indirect vector-mediated horizontal transmission is expected to lead to an increase in pathogen virulence because it facilitates transmission, or partially dissociates host longevity from transmission, and thereby changes the relationship between virulence and transmission [12,13]. Over ecological time, in contrast, a change in mode of horizontal transmission from direct to indirect, vector-mediated, might have an immediate effect in elevating virulence, e.g., through an increase in inoculum size [14] or evasion of the host immune response [15]. The varroa mite (*Varroa destructor*) is an illuminating example of this phenomenon. It is an obligate honey bee ectoparasite that attacks adults (workers, drones, and queens) as well as brood [16]. It has had significant impacts on honey bee colony health, in part as a consequence of its role as a transmitter of a cocktail of viruses, particularly *Deformed wing virus* (DWV), while feeding on honey bee hemolymph and fat bodies [17,18]. The mite effectively transforms relatively benign, directly transmitted viruses into highly virulent, indirectly transmitted (vector-mediated) viruses. The case of DWV is exemplary [6,19], with populations of honey bees invaded by varroa mites that transmit DWV leading to extremely high viral prevalence, intensity of infection (viral titer) and colony collapse on Hawaii [20] and New Zealand [21].

Honey bees possess natural defense mechanisms to reduce the effects of their pathogens. Such mechanisms include pathogen resistance, building infection barriers or mounting protective responses once infection occurs using mechanical, physiological and immune defenses [7]. Mechanical barriers include insect cuticle and epithelial membranes, which often prevent entry of microbes. Physiological inhibitors to microbial invasion may include changes in, for example, the pH of the insect gut [22,23]. Immune defense includes multiple innate immune signaling pathways (e.g., Toll, Imd, JAK-STAT, JNK) that are thought to play diverse defensive, including antiviral, roles in insects [24,25,26]. However the most widespread [27] and robust defenses of insects against viruses are the RNA interference (RNAi) pathways [28,29,30] that honey bees employ in response to infection by the highly virulent *Israeli acute paralysis virus* (IAPV) [31] and likely other viruses.

One of the most prevalent honey bee viruses is *Black queen cell virus* (BQCV) [32,33,34], so-called because it kills developing queen honey bee larvae, whose necrotic remains blacken their pupal cells [35,36,37]. Nurse honey bees horizontally transfer BQCV from infected cells to healthy larvae in brood food [38], and queens may also vertically transmit it to eggs [9]. BQCV is also associated with co-infection by the Microsporidian *Nosema apis* [39]. Experimental feeding of BQCV to larvae arrested development and caused mortality in a dose-dependent fashion [40], whereas 2-day old adult workers fed 1.4 × 10^9^ genome equivalents of BQCV lived as long as controls [39] and merely exhibited up-regulation of several genes of the RNAi pathway, including *Dicer-like* and *Argonaut 2* (*AGO2*) [41]. Though infected adults remain asymptomatic [9], BQCV has been found at high prevalence and titer in collapsing colonies [21], suggesting that its mode of transmission may not only be direct via ingestion but may also include indirect, vector-mediate transmission, e.g., by varroa mites.

Here, using a controlled laboratory experimental design, we tested the effect of mode of horizontal transmission of BQCV on its virulence in adult honey bees by inoculating hosts by feeding, replicating direct (fecal/food-oral) transmission, or by injection, replicating vector-mediated, indirect transmission. As a measure of virulence, we recorded worker survival and, in addition, quantified the intensity of infection (viral titer per bee) and innate immune gene expression. We hypothesized that injection (analogous to indirect, vector-mediated horizontal transmission) by BQCV would lead to far higher virulence and greater immune response than direct, fecal/food-oral transmission. Our principal result, that vector-based transmission elevated BQCV virulence, has implications for the health of honey bees where varroa mites are nowadays endemic.

## 2. Materials and Methods

### 2.1. Honey Bees

Honey bee *(A*. *mellifera carnica*) colonies maintained in the General Zoology apiary at Martin Luther University Halle-Wittenberg, Germany, were treated to control varroa mites with Bayvarol^®^ strips in November 2018. In July 2019, colonies were screened by reverse transcription and quantitative PCR (qPCR) for seven common viral targets: DWV genotypes A and B, BQC, the *Acute bee paralysis virus* complex (ABPV, targeting *Acute bee paralysis virus, Israeli acute paralysis virus* and *Kashmir bee virus*), *Chronic bee paralysis virus* (CBPV), *Sacbrood virus* (SBV) and *Slow bee paralysis virus* (SBPV), using the primers listed in Appendix A (Table A1) and methods described in [42] and outlined in Section 2.3 below. Viruses were not detected in colonies at a threshold cycle (Ct) of 35, a threshold that minimizes the rate of false positives [43], suggesting that they were largely free of viral pathogens.

### 2.2. BQCV Inoculation

Aliquots of BQCV, a positive single stranded RNA (+ssRNA) virus (family Dicistroviridae), were obtained from a propagated inoculum [32]. Inocula contained only the viral target and had no trace other common honey bee RNA viruses, determined by qPCR as described in [42] and outlined in Section 2.3 below. Viral inocula were firstly diluted in 0.5 M cold potassium phosphate buffer (PPB) (pH 8.0) to final concentrations of 10^8^ genome equivalents per µL, quantified by qPCR as described in [42] and outlined in Section 2.3 below.

Freshly emerged honey bees from one virus-free colony were exposed to BQCV either by feeding or injection to test the mode of horizontal transmission on viral virulence. We transferred a single frame with sealed worker brood to an incubator at 35 °C and 80% relative humidity (RH). The next day, we collected 360 newly emerging bees and randomly assigned them to three groups (control, BQCV-feeding and BQCV-injection). For the BQCV-feeding treatment, 10 µL of 50% sugar solution containing 10^8^ BQCV was fed using a micropipette to individual bees that had been starved for 2 h, but without prior anesthesia. BQCV fed to adult honey bees has been shown to lead to an increase in viral titer [44]. The dose we used approximates that required to kill a honey bee larva but not an adult [40] yet sufficient to induce a transcriptome response in adult bees after 13 days [41]. For the BQCV-injection treatment, bees were chilled on ice for 3 min, and then, one µL of viral inoculum containing 10^8^ BQCV was injected into a bee directly into its hemolymph between its second and third abdominal tergites using a Hamilton syringe (hypodermic needle outer diameter: 0.235 mm).

In order to control for the effect of either injection or feeding during treatments, control and BQCV-injection groups were also fed 10 µL of 50% sugar solution devoid of virus and, similarly, control and BQCV-fed groups were injected with one µL of buffer (PPB) devoid of virus. We afterwards kept bees in metal cages (10 × 10 × 6 cm) containing an 8 cm^2^ piece of organic beeswax following standard methods [45]. Each cage contained 30 newly emerged worker bees of the same treatment and was provided with sugar water ad libitum.

Cages were placed into incubators at 30 ± 1 °C and 50% RH. Four replicate cages where used for each treatment. Mortality was recorded daily for 10 days as our measure of virulence. Then we collected all surviving bees in all cages and froze them at −80 °C prior to quantifying viral load and gene expression, as described below.

### 2.3. RNA Extraction and Detection of Virus

When testing whether honey bee source colonies were free of an RNA virus infection, we collected 10 adult worker honey bees per colony from the brood nest, crushed them in a plastic RNAse-free mesh bag (BioReba, Reinach, Switzerland) with five mL of RLT-buffer after snap-freezing them on dry ice, and then recovered 100 µL of homogenate. BQCV titers in adult worker bees arising from inoculation experiments were determined by crushing whole bees individually (8 bees per treatment) in 500 µL of RLT-buffer (with 1% β-mercaptoethanol) using a plastic pestle, of which 100 µL were used for RNA isolation. RNA was extracted from bee homogenates using an RNeasy mini kit (Qiagen, Hilden, Germany) following the manufacturer’s instructions in a QiaCube robot (Qiagen). cDNA was synthesized from RNA extracts using oligo(dT)_18_ primers (Thermo Fisher Scientific, Schwerte, Germany) and reverse transcriptase (M-MLV and Revertase, Promega, Mannheim, Germany) following the manufacturer’s instructions. For cDNA synthesis, 800 ng of RNA were used, after which the resultant cDNA was diluted 1:10 prior to use in qPCR.

Real-time or quantitative PCRs (qPCRs) were performed on a Bio-Rad C1000 thermal cycler using SYBRgreen Sensimix (Bioline, Luckenwalde, Germany) and the BQCV primers listed in Appendix A (Table A1) to quantify BQCV titer in experimental bees. We simultaneously amplified β-actin (AMActin/Actin related protein 1, primers listed in Appendix A, Table A2), a reference (housekeeping) gene for *A. mellifera*, as a check on successful RNA extraction and cDNA synthesis. Amplification steps were 5 min at 95 °C, followed by 40 cycles of 10 s at 95 °C and 30 s at 57 °C (including a read at each cycle). Following qPCR, DNA was denatured for 1 min at 95 °C and then cooled to 55 °C in 1 min, and a melting profile was obtained from 55 to 95 °C at a 0.5 °C increment per second to confirm that the correct PCR product had been amplified. Absolute quantification of virus was calculated using a standard curve generated with ten 10-fold dilutions of the BQCV purified PCR product, as described in [42]. To minimize the rate of false positives of the virus, a Ct of 35 cycles was set [43]. We simultaneously screened the same bees for six other viral targets (the ABPV complex, DWV-A, DWV-B, CPBV, SBV, SBPV) to check on co-infection with other virus. Bees were largely devoid of these other viruses (Supplementary Materials).

### 2.4. Gene Expression

We used qPCRs to quantify effects of a change in mode of horizontal transmission of BQCV on the expression of three key innate immune genes with well documented involvement in the insect anti-viral response, namely *Dicer-like*, *Argonuate 2 (AGO2)* and *Tarbp2-like*, which are all part of the honey bee RNAi pathway [31,46]. Primers were taken from [31,46] (Appendix A, Table A2). To quantify gene expression, we used the same cDNA synthesized for viral quantification (Section 2.5), and qPCRs were performed on the same machine following the same protocols used for viral detection, with slight modification in amplification steps (10 min at 95 °C instead of 5 min in viral detection). qPCR was followed by a dissociation step to validate that only a single product was amplified in each reaction. For each target gene, the abundance of transcripts was quantified according to the Mean Normalized Expression (MNE) method of [47], with β-actin used as a reference (housekeeping) gene. We also determined primer efficiency using standard curves of serial dilutions of cDNA. We confirmed acceptable reaction conditions for all genes, with coefficients of determination (*R*^2^) > 0.99 and efficiencies between 101% and 104% (Appendix A, Table A2).

### 2.5. Statistical Analysis

All statistical analyses and data visualizations were performed in GraphPad Prism 7.0 (GraphPad, La Jolla, CA, USA). To compare survival between treatments we used Kaplan–Meier log-rank paired tests. To compare BQCV titers in bees exposed to virus by either feeding or injection, we used Student’s *t*-test on log_10_ transformed absolute viral titers. Effects on gene expression between treatments were analyzed by one-way ANOVA followed by Tukey’s post hoc tests. An alpha level of 0.05 was used to define significance for all tests.

## 3. Results

### 3.1. Virulence

**Figure 1:**
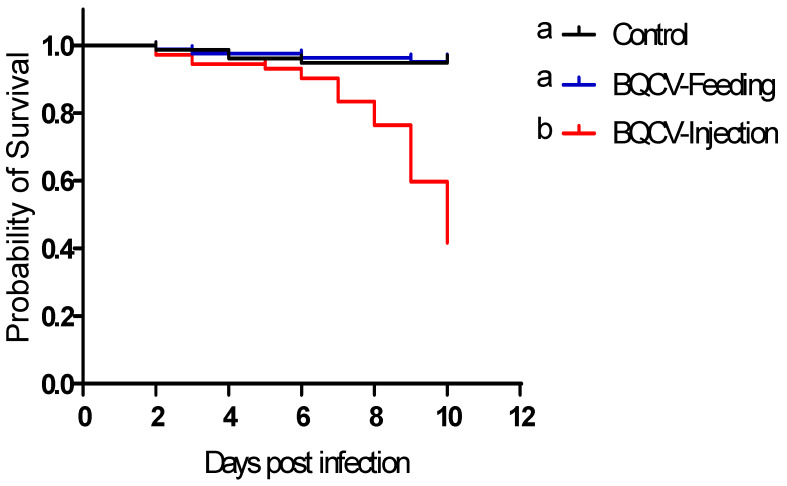
Survival of honey bees treated with Black queen cell virus (BQCV). Bees were either injected with one µL inoculum containing 10^8^ BQCV or fed 10 µL of sugar syrup containing 10^8^ BQCV on day 0. Controls received the same treatments devoid of virus. Different lower case letters indicate statistically significant differences between treatments (Log-rank (Mantel-Cox), *p* < 0.05).

Inoculating adult honey bees with BQCV by injection significantly reduced their survival (61% dead after 10 days, median time to death: 10 days) as compared to controls and to those inoculated orally with BQCV (5.6% dead after 10 days; log-rank (Mantel Cox) paired test, *p* < 0.0001). There was no significant difference in survival of bees that were inoculated orally compared to control bees (log-rank (Mantel Cox) paired test, *p* > 0.05) (Figure 1).

### 3.2. Effects on Viral Titer

**Figure 2:**
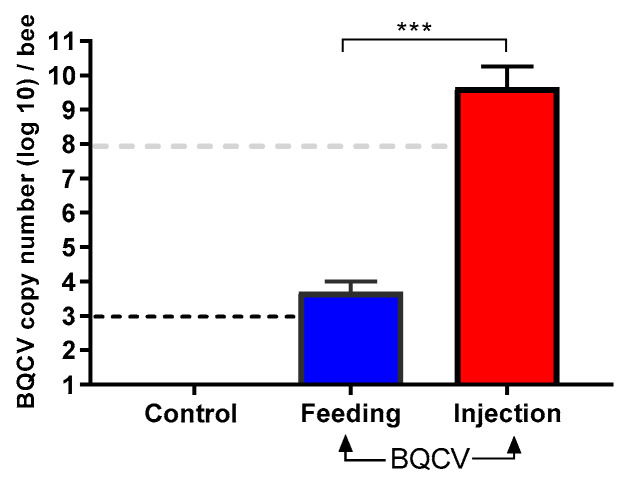
Absolute quantification (mean ± SD) of BQCV per adult honey bee at 10 days post inoculation. Bees (*n* = 8 bees per treatment) either injected with one µL inoculum containing 10^8^ BQCV or fed 10 µL of sugar syrup containing 10^8^ BQCV. Controls received the same treatments devoid of virus. There was a significant difference in BQCV load (genome equivalents, log_10_-transformed) between fed and injected bees (Student’s *t*-test, *** *p* < 0.001). Black dashed line indicates the threshold of detection while the gray dashed line indicates the initial dose of BQCV.

BQCV-injected bees had a significantly higher viral titer at 10 days post inoculation than bees that were orally inoculated with the virus (BQCV-injected, mean titer: 7.68 × 10^9^ (95% CI 2.4 × 10^9^–1.3 × 10^10^) BQCV genome equivalents; BQCV-fed, mean titer: 6.18 × 10^3^ (95% CI 2.5–9.8 × 10^3^) BQCV genome equivalents; Student’s *t*-test, *p* < 0.001). Bees that were inoculated by feeding contained over four orders of magnitude less than the initial dose of 10^8^ BQCV at 10 days after feeding. Control honey bees remained devoid of BQCV (Figure 2), after having been both fed and injected at day zero with control solutions devoid of virus.

### 3.3. Effects on Gene Expression

**Figure 3:**
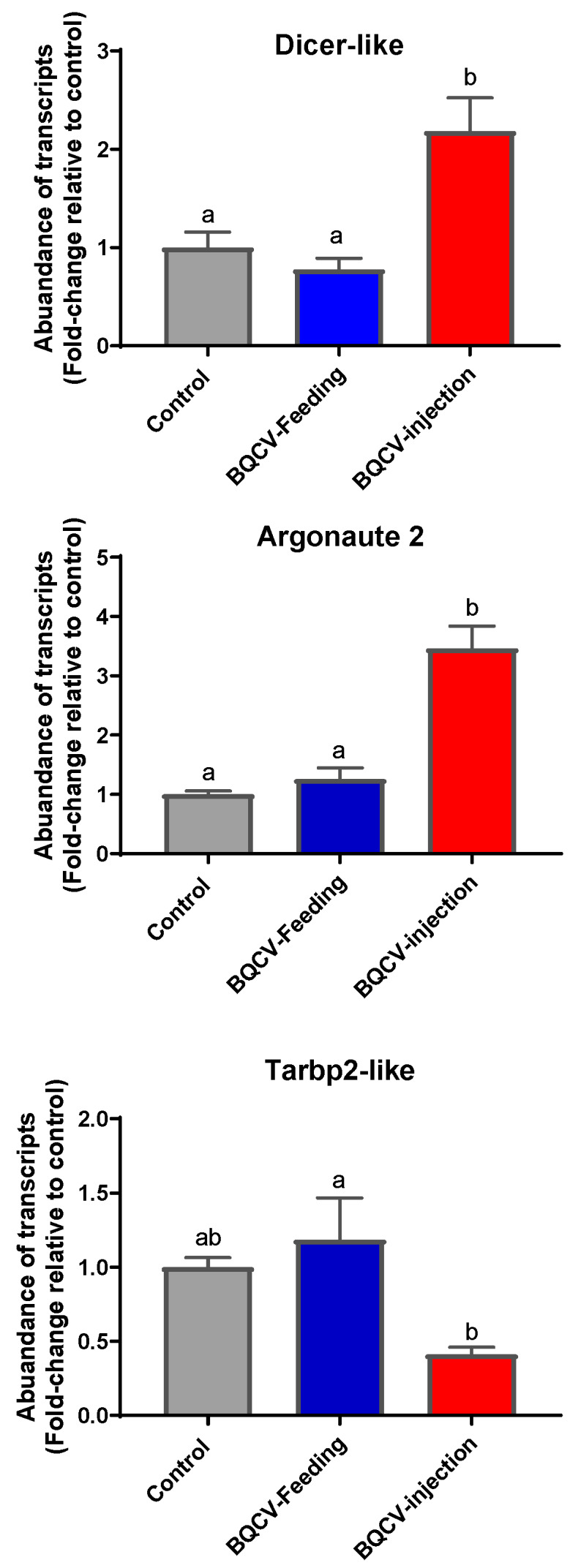
Fold-change (mean ± SE) of transcripts of three innate immune pathway genes in adult honey bees at 10 days post BQCV inoculation. Bees (*n* = 6 bees per treatment) were either injected with one µL inoculum containing 10^8^ BQCV or fed 10 µL of sugar syrup containing 10^8^ BQCV. Controls received the same treatments devoid of virus. Different lower case letters indicate statistically significant differences between treatments (one-way ANOVA with Tukey’s post-hoc test, *p* < 0.05).

*Dicer-like* and *Argonuate 2* (AGO2) gene transcripts were significantly upregulated in BQCV-injected bees compared to both BQCV-fed bees and to control bees (*p* < 0.05, Figure 3). The expression of *Tarbp2-like* was significantly downregulated in BQCV-injected bees compared to BQCV-fed bees (ANOVA *p* < 0.05). Importantly, differences between bees experimentally infected with BQCV (by feeding or by injection) and controls were not statistically significant (*p* > 0.05; see Figure 3).

## 4. Discussion

We found that injecting BQCV directly into adult honey bees, analogous to vector-mediate or indirect horizontal transmission, resulted in their much earlier death, increased viral load and changed expression of RNAi pathway genes. In contrast, adult bees fed BQCV did not exhibit elevated mortality to inoculation, as also noted earlier [39,40].

We hypothesized that injection of BQCV into adult honey bees would result in much greater virulence than oral transmission. Our results support this hypothesis, which may represent a general phenomenon in honey bees and possibly other insects in response to BQCV and other viral pathogens. For example, [48] also found that injection or vector-mediated transmission of DWV led to far higher viral titers in honey bees than feeding DWV. In *Drosophila melanogaster*, oral transmission with *Drosophila C virus* causes mild infection whereas injection causes rapid host death [49,50].

Dose size is unlikely to explain our results because the same quantity of BQCV was inoculated by feeding and by injection. A caveat is that we cannot tell how many virions reached target host cells by injection *versus* feeding; oral infection might entail a far greater bottleneck for BQCV than injection into the haemocoel.

One possible explanation for our results is that injection or vector mediated transmission circumvents the protective barrier of the digestive system because virus particles directly enter the bee’s hemocoel rather than having to pass through the gut lumen [23]. Adult honey bees possess diverse mechanisms to mitigate the effects of ingested pathogens that include changes in the pH of the honey bee gut lumen and production of inhibitory substances [22,23]. For example, extracts of adult midguts inhibited the development of *Paenibacillus larvae* bacteria, the causative agent of American foulbrood disease [22]. Honey bee gut microbiota may also play a role in inhibiting viral pathology [51,52]. These mechanisms could explain why bees that were inoculated by feeding contained a lower viral titer than the initial dose of 10^8^ BQCV genome equivalents, as also noted earlier [39].

The difference in virulence of BQCV associated with mode of horizontal transmission that we found in adult honey bees (vector-mediated: high virulence; direct, per os: low virulence) is also seen in juvenile stages. Honey bee larvae show a dose-dependent response to BQCV when fed virus; doses of 10^4^ and 10^7^ caused little appreciable mortality to larvae whereas 10^9^ was necessary to cause significant mortality [40]. In contrast, BQCV exhibits high virulence when injected into pupae [36,53,54], just as we found in adults. The larval honey bee gut may also represent a barrier to pathogens such as BQCV, just as it seems to in adults.

BQCV is a highly prevalent virus of honey bees in Europe and Asia [55,56,57,58]. Its prevalence in honey bees across Vermont, USA, was recently found to be 100%, with titers per bee ranging from 10^6^ to 10^9^ [59]. Nevertheless, despite its high prevalence, BQCV is typically found in the colony as a covert infection without causing noticeable symptoms and does not appear to be associated with colony decline [60], suggesting that it is typically transmitted horizontally per os but not regularly vectored, e.g., by varroa mites.

RNAi pathways are considered the most robust antiviral defense mechanisms to restrict viral replication in honey bees [3,6]. The best characterized RNAi pathway produces small non-coding RNAs, known as small interfering RNAs (siRNAs), that interact with *argonaute* proteins to detect and destroy viral (or endogenous) double-stranded (ds) RNAs via the RNA-induced silencing complex (RISC) in a sequence-specific manner [61]. In BQCV-injected bees (but not in BQCV-fed bees) we found that two RNAi pathway genes were up-regulated: *Argonaute 2 (AGO2)* and *Dicer-like*, suggesting that bees were mounting a response to viral challenge, though it is not possibly to determine whether the response was successful or not in limiting viral proliferation. These differences in response of BQCV injected versus BQCV fed adults could be primarily due to the high viral titer within bees at 10 days post BQCV-injection. Alternatively, BQCV-fed bees might employ additional responses to viral challenge. Similar upregulation of *Argonaute 2* (*AGO2*) and *Dicer-like* have been also reported for honey bee adults when fed a slightly higher dose of BQCV (1.4 × 10^9^ genome equivalents) than we used [41] or when fed an unquantified inoculum of IAPV [31] that, like BQCV, is a +ssRNA virus of the family Dicistroviridae.

Given the importance of RNAi pathways in limiting broad classes of viruses, it is not surprising that various insect RNA and DNA viruses have evolved strategies to counter host RNAi responses. Virus-encoded suppressors of RNAi (VSR) can inhibit the RNAi pathway and protect viral dsRNA replication intermediates from *dicer-2* cleavage [62]. *Dicer*, TAR RNA-binding protein (*tarbp*) and *scavenger receptor class Cs* (SCR-C) are all VSR-targeted proteins whose mRNA expression can be modulated by viral infection [63]. *Tarbp* is a co-factor of *dicer* that functions as part of the RISC [63]. We found that *Tarbp2-like* gene expression was significantly down-regulated in BQCV-injected bees compared to BQCV-fed bees. This suggests that BQCV might induce the expression of VSRs at high BQCV titers that inhibit *Tarbp* expression and disrupt RISC mediated RNAi. This could be a reason for the high mortality we found in BQCV-injected bees as compared to BQCV-fed bees. IAPV-fed adults honey bees, in contrast, exhibited up-regulation of *Tarbp2-like* [31]. Future studies are required to investigate the multi-faceted mechanisms by which these VSRs induced by BQCV (and potentially other viruses such as IAPV) inhibit host RNAi pathways and promote viral replication, with correspondingly high virulence.

In conclusion, a change in the mode of experimental transmission of BQCV from fecal/food-oral (direct horizontal) to vector-mediate (indirect horizontal) resulted in a dramatic increase in mortality and viral titer of adult honey bees. If BQCV transmission became vector-mediated in the hive, it might lead to adverse effects on the health of honey bee colonies. Collapsing colonies containing high numbers of varroa mites and rising BQCV titers might then speed up colony collapse by vectoring BQCV to pupae and adults. The ability of varroa mites to transmit BQCV and the frequency with which they do so in declining colonies deserve closer scrutiny.

## Supplementary Materials

The following are available online at https://www.mdpi.com/1999-4915/12/5/535/s1, Excel file providing (Sheet 1) mortality data, (Sheet 2) viral titers and (Sheet 3) gene expression Ct values.

## Appendix A

**Table A1:** List of primers used for quantification of viruses by reverse transcription followed by qPCR and efficiency of qPCR.

Viral Target	Primer Name	Sequence	qPCR Efficiency	Reference
ABPV *	KIABPV-F6648	CCTTTCATGATGTGGAAAC	---	[64]
	KIABPV-B6707	CTGAATAATACTGTGCGTATC		
BQCV	BQCV-qF7893	AGTGGCGGAGATGTATGC	103.3%	[65]
	BQCV-qB8150	GGAGGTGAAGTGGCTATATC		
CBPV	CBPV1-qF1818	CAACCTGCCTCAACACAG	90.2%	[65]
	CBPV1-qB2077	AATCTGGCAAGGTTGACTGG		
DWV-A	DWV-F8668	TTCATTAAAGCCACCTGGAACATC	85.6%	[66]
	DWV-B8757	TTTCCTCATTAACTGTGTCGTTGA		
DWV-B	VDVq-F2	TAT CTT CAT TAA AAC CGC CAG GCT	86.0%	[32]
	VDVq-R2a	CTT CCT CAT TAA CTG AGT TGT TGTC		
SBPV	SBPV-F3177	GCGCTTTAGTTCAATTGCC	93.8%	[67]
	SBPV-B3363	ATTATAGGACGTGAAAATATAC		
SBV	SBV-qF3164	TTGGAACTACGCATTCTCTG	92.1%	[65]
	SBV-qB3461	GCTCTAACCTCGCATCAAC		

ABPV *, primers amplify Acute bee paralysis virus, Israeli acute paralysis virus and Kashmir bee virus.

**Table A2:** List of primers used for quantification of transcripts in honey bees by reverse transcription followed by qPCR and efficiency of qPCR.

Locus	Category	Sequence	qPCR Efficiency	Reference/Accession Number
*Dicer-like*	Innate immunity	F: CCAACAGGAGCTGGAAAAAC	103%	[31] XM_006571316.1
		R: TCTCCACTAAGTGCTGCACAA		
*Argonaute 2* (*AGO2*)	Innate immunity	F:TCAACAGCAGCAATCGGATA	104%	[31] XM_395048.5
		R:TTGCGGTGAACTTTGTTGTT		
*Tarbp2-like*	Innate immunity	F: AGGGTTTGCCACATGAAAGA	102%	[31] XM_006564411.1
		R: AATCAGCATAACGGGCACTC		
AMActin (*β-actin*)	Housekeeping	F: ATGCCAACACTGTCCTTTCTGG	101%	[66]
		R: GACCCACCAATCCATACGGA		
